# Clinical efficacy of Linggui Tanxue acupuncture combined with extracorporeal shock wave therapy for piriformis syndrome: a prospective comparative clinical study

**DOI:** 10.3389/fmed.2026.1897807

**Published:** 2026-07-31

**Authors:** Chao Xue, Jie Ma, Longlong Wei, Songgang Liu

**Affiliations:** Qingdao Huangdao District Central Hospital, Qingdao, Shandong, China

**Keywords:** acupuncture, extracorporeal shock wave therapy, musculoskeletal ultrasound, pain, piriformis syndrome

## Abstract

**Objective:**

To evaluate the short-term clinical efficacy and safety of Linggui Tanxue acupuncture combined with extracorporeal shock wave therapy (ESWT) in patients with piriformis syndrome (PS).

**Methods:**

Sixty patients with PS aged 35 to 45 years were prospectively allocated using a random-number table into a control group receiving ESWT alone and a combined-treatment group receiving Linggui Tanxue acupuncture followed by ESWT, with 30 patients in each group. The treatment course lasted 2 weeks. Visual analogue scale (VAS) pain scores, musculoskeletal ultrasound (MSUS)-measured piriformis muscle thickness, clinical efficacy category, and adverse events were evaluated. Pain assessment and ultrasound measurement were performed by assessors blinded to treatment allocation. Because the study was not prospectively registered and no predefined statistical analysis plan or formal sample size calculation was established before initiation, the analysis should be interpreted as exploratory.

**Results:**

Baseline sex distribution, age, disease duration, VAS scores, and piriformis muscle thickness were comparable between groups (all *p* > 0.05). After 2 weeks, both groups showed significant reductions in VAS scores and piriformis muscle thickness (both *p* < 0.001). The combined-treatment group showed significantly greater improvement than the ESWT-alone control group in the two continuous outcomes: post-treatment VAS score was lower in the combined-treatment group (1.44 ± 0.10 vs. 3.57 ± 0.17; mean difference, −2.12; 95% CI, −2.20 to −2.05; *p* < 0.001), and post-treatment piriformis thickness was also lower (9.04 ± 0.16 mm vs. 12.16 ± 0.25 mm; mean difference, −3.13 mm; 95% CI, −3.23 to −3.02; *p* < 0.001). The combined-treatment group also had a significantly higher total effective rate than the control group (86.7% vs. 46.7%; Fisher exact *p* = 0.002), but this categorical outcome was considered supportive and should be interpreted cautiously. No serious adverse events occurred.

**Conclusion:**

Linggui Tanxue acupuncture combined with ESWT may provide greater short-term pain relief and greater ultrasound-measured improvement in piriformis muscle thickness than ESWT alone in patients with PS. The findings are encouraging but not definitive because of the absence of prospective registration, lack of a predefined statistical analysis plan, lack of formal sample size calculation, and the short 2-week follow-up period. Larger prospectively registered multicenter studies with predefined statistical analysis plans, formal sample size calculations, and longer follow-up are warranted.

## 1 Introduction

Piriformis syndrome (PS) is a neuromuscular condition characterized by buttock pain with possible radiation along the sciatic nerve distribution. It is generally associated with irritation or compression of the sciatic nerve by the piriformis muscle, which may occur in the setting of muscle spasm, local inflammation, edema, fibrosis, or fascial adhesion. Patients commonly present with unilateral hip or buttock pain, radiating lower-limb pain or numbness, tenderness over the piriformis projection area, and pain aggravated by sitting, walking, or hip movement (1, 2).

Conservative management is usually considered the initial therapeutic approach for PS and may include physical therapy, analgesic medication, stretching, local injection, acupuncture, and extracorporeal shock wave therapy (ESWT) (1, 3–5). However, symptom relief may be incomplete or delayed in some patients, and recurrence is not uncommon. Therefore, combined interventions targeting both pain modulation and local soft-tissue dysfunction may have clinical value.

Linggui Tanxue acupuncture is a targeted acupuncture technique that identifies tender points, tendon knots, and meridian-sensitive reaction points along the lesion area (6, 7). This approach aims to relieve local spasm and improve regional circulation. ESWT is a non-invasive rehabilitation modality that may reduce pain and improve soft-tissue metabolism through mechanical stimulation (3–5, 8, 9). Musculoskeletal ultrasound (MSUS) can objectively display the morphology and thickness of the piriformis muscle and may provide a quantitative imaging indicator for diagnosis and treatment evaluation in PS (10–14). Therefore, this study evaluated the short-term clinical efficacy and safety of Linggui Tanxue acupuncture combined with ESWT for PS using VAS score and MSUS-measured piriformis muscle thickness as the main outcomes.

## 2 Materials and methods

### 2.1 Study design and participants

This was a prospective comparative clinical study conducted in the Department of Rehabilitation Medicine, Qingdao Huangdao District Central Hospital. A total of 60 patients with PS admitted from March 2024 to December 2025 were enrolled and allocated into a control group and a combined-treatment group using a random-number table, with 30 patients in each group. The control group received ESWT alone, whereas the combined-treatment group received Linggui Tanxue acupuncture plus ESWT. The treatment duration was 2 consecutive weeks.

Because this study was not prospectively registered and no predefined statistical analysis plan (SAP) was established before initiation, it is reported as a prospective comparative clinical study rather than as a fully registered interventional trial. No formal *a priori* sample size calculation was performed. The sample size reflected the number of eligible patients available during the study period and was not based on a predefined power analysis. Accordingly, the study should be considered exploratory and hypothesis-generating, and the precision of treatment-effect estimates should be interpreted with caution.

This study was approved by the Ethics Committee of Qingdao Huangdao District Central Hospital (Approval No.: HDZY-2024-012). Written informed consent was obtained from all participants before enrollment. This study was conducted in accordance with the Declaration of Helsinki. The lack of prospective registration, absence of a predefined SAP, and absence of formal power calculation are acknowledged as important methodological limitations.

### 2.2 Diagnostic assessment and eligibility criteria

PS was diagnosed clinically using a combination of symptoms, physical findings, and exclusion of alternative causes. Eligible patients had unilateral hip or buttock soreness or distending pain, radiating pain or numbness along the sciatic nerve distribution, localized tenderness in the projection area of the piriformis muscle, pain aggravated by sitting, walking, or hip movement, and limited external rotation or abduction of the hip joint (1, 2). Because there is no universally accepted diagnostic gold standard for PS, patient selection was based on concordant clinical features rather than a single diagnostic test.

Alternative causes of buttock and radiating leg pain were excluded through medical history, physical and neurological examination, hip and sacroiliac joint assessment, and review of available imaging or electro diagnostic information when clinically indicated. Patients were excluded if findings suggested lumbar disc herniation, lumbar spinal stenosis, sacroiliac joint disorder, femoral head disease, hip joint disease, lumbosacral radiculopathy, or other causes of low back and leg pain.

Inclusion criteria were as follows: (1) meeting the clinical diagnostic criteria for PS described above; (2) aged 35 to 45 years; (3) normal communication ability and ability to complete treatment and scale evaluation; and (4) written informed consent provided by the patient. Exclusion criteria were as follows: (1) lumbar disc herniation, lumbar spinal stenosis, sacroiliac joint disorder, femoral head disease, or other causes of low back and leg pain; (2) hip skin ulceration, dermatitis, infection, or contraindications to acupuncture or ESWT; (3) coagulation dysfunction, severe cardiovascular or cerebrovascular disease, abnormal liver or kidney function, or mental illness; (4) pregnancy or lactation; and (5) receipt of local block, acupotomy, surgery, or other related treatment within the previous month.

### 2.3 Interventions

#### 2.3.1 Control group: ESWT alone

The control group received ESWT alone and did not receive acupuncture. Tender points, spasm nodules, and pain radiation points in the piriformis region were marked as treatment targets. The ESWT parameters were as follows: impact frequency 10–15 Hz, energy density 0.15–0.25 mJ/mm2, local fixed-point treatment combined with regional scanning, and 1,500–2000 impulses per session. Treatment was performed 3 times per week for 2 weeks. All ESWT procedures were performed using the same standardized protocol by rehabilitation physicians with more than 5 years of clinical experience.

#### 2.3.2 Combined-treatment group: Linggui Tanxue acupuncture plus ESWT

Patients in the combined-treatment group received Linggui Tanxue acupuncture followed by ESWT. Patients were placed in the prone or healthy lateral decubitus position with the affected hip fully exposed. After routine skin disinfection with povidone-iodine, Huantiao (GB30), Zhibian (BL54), and Baihuanshu (BL30) were selected as basic acupoints. The operator palpated along the piriformis muscle region from shallow to deep and from light to heavy pressure to identify tender sensitive points, tendon knots, and cord-like positive reaction points as targeted stimulation sites. Disposable sterile filiform needles (0.30 mm × 50–75 mm) were inserted vertically or obliquely according to the anatomical level. After the needle reached the muscle layer, twisting and lifting-thrusting manipulations were performed until deqi was achieved, manifested as local soreness, distension, heaviness, or radiating sensation. Needles were retained for 20 min. ESWT was then performed 30 min after acupuncture using the same target-marking method, parameters, frequency, and treatment course as the control group.

### 2.4 Outcome measures

*Pain score*: The VAS was used to evaluate pain before treatment and after 2 weeks of treatment. The scale ranged from 0 to 10 points, with higher scores indicating more severe pain. Pain scores were collected by an independent evaluator blinded to treatment allocation to minimize assessment bias (15).

*Piriformis muscle thickness*: MSUS was used to measure the thickest part of the affected piriformis muscle before treatment and after 2 weeks of treatment. All ultrasound examinations were performed by the same senior ultrasonographer who was blinded to group allocation. The same anatomical landmarks, scanning plane, patient positioning, and measurement protocol were used for all patients and for both pre-treatment and post-treatment assessments (11–14). Formal intra-observer and inter-observer reliability testing was not performed; therefore, measurement reproducibility was addressed through standardization and assessor blinding rather than through a separate reliability analysis.

*Clinical efficacy*: Clinical efficacy was classified as cure, markedly effective, effective, or ineffective according to the Criteria for Diagnosis and Therapeutic Effect Evaluation of Traditional Chinese Medicine Syndromes (16). Total effective rate was calculated as (cured + markedly effective + effective cases)/total cases × 100%. Because this categorical outcome is not widely used in international musculoskeletal research, it was treated as a supportive secondary outcome and interpreted together with the validated pain outcome and objective ultrasound findings.

*Safety*: Adverse events, including subcutaneous ecchymosis, local pain, skin redness or swelling, dizziness, nausea, hematoma, infection, and nerve injury, were recorded throughout the treatment period.

### 2.5 Statistical analysis

SPSS 26.0 software was used for data analysis. Continuous variables were expressed as mean ± standard deviation. Normality was assessed using the Shapiro–Wilk test before parametric testing. Paired t-tests were used for within-group comparisons before and after treatment, and independent-sample t-tests were used for between-group comparisons when parametric assumptions were reasonably met. For ordinal outcomes or variables showing deviation from normality, Wilcoxon signed-rank tests or Mann–Whitney U tests were additionally performed as sensitivity analyses. Mean differences and 95% confidence intervals (95% CI) were calculated for major continuous outcomes. Categorical variables were expressed as cases and percentages and analyzed using chi-square or Fisher exact tests as appropriate. A two-sided *p* value < 0.05 was considered statistically significant. Because no predefined SAP was available, inferential analyses should be viewed as exploratory, and emphasis was placed on effect direction, effect size, and confidence intervals rather than on *p* values alone.

## 3 Results

### 3.1 Baseline characteristics

A total of 60 patients aged 35 to 45 years were included, with 30 patients in each group. Mean age was comparable between the control group and combined-treatment group (40.33 ± 2.90 years vs. 40.10 ± 2.67 years; *t* = 0.32, *p* = 0.747). The sex distribution was identical in the two groups (male/female: 15/15 vs. 15/15; *p* = 1.000). Disease duration was also comparable between the control group and combined-treatment group (10.60 ± 5.73 months vs. 11.03 ± 5.90 months; *t* = −0.29, *p* = 0.774). Baseline VAS score and piriformis muscle thickness were similar between the groups (both *p* > 0.05). Detailed baseline characteristics are presented in Table 1.

**Table 1:** Baseline characteristics of the two groups.

Variable	Control group (*n* = 30)	Combined-treatment group (*n* = 30)	Statistic	*P* value
Age, years	40.33 ± 2.90	40.10 ± 2.67	*t* = 0.32	0.747
Male/Female	15/15	15/15	*χ*^2^ = 0.00	1.000
Disease duration, months	10.60 ± 5.73	11.03 ± 5.90	*t* = −0.29	0.774
Pre-treatment VAS score	7.28 ± 0.20	7.21 ± 0.15	*t* = 1.55	0.126
Pre-treatment piriformis thickness, mm	17.01 ± 0.32	16.86 ± 0.27	*t* = 1.99	0.051

Values are shown as mean ± standard deviation or number of cases.

### 3.2 VAS pain scores before and after treatment

Before treatment, there was no statistically significant difference in VAS scores between the control group and combined-treatment group (7.28 ± 0.20 vs. 7.21 ± 0.15; *t* = 1.55, *p* = 0.126). After 2 weeks of treatment, VAS scores decreased significantly in both groups (both *p* < 0.001). The post-treatment VAS score was significantly lower in the combined-treatment group than in the control group (1.44 ± 0.10 vs. 3.57 ± 0.17; mean difference, −2.12; 95% CI, −2.20 to −2.05; *p* < 0.001). Nonparametric sensitivity analysis for VAS outcomes showed the same direction of effect and remained statistically significant after treatment. VAS results are presented in Table 2.

**Table 2:** Comparison of VAS pain scores between the two groups before and after treatment.

Group	*n*	Pre-treatment VAS	Post-treatment VAS	Within-group *P*	Between-group *P* after treatment
Control group	30	7.28 ± 0.20	3.57 ± 0.17	<0.001	
Combined-treatment group	30	7.21 ± 0.15	1.44 ± 0.10	<0.001	<0.001

### 3.3 Piriformis muscle thickness before and after treatment

Before treatment, piriformis muscle thickness was comparable between the control group and combined-treatment group (17.01 ± 0.32 mm vs. 16.86 ± 0.27 mm; t = 1.99, *p* = 0.051). After treatment, piriformis muscle thickness decreased in both groups (both *p* < 0.001). The post-treatment thickness was significantly lower in the combined-treatment group than in the control group (9.04 ± 0.16 mm vs. 12.16 ± 0.25 mm; mean difference, −3.13 mm; 95% CI, −3.23 to −3.02; *p* < 0.001). Nonparametric sensitivity analysis confirmed a significant between-group difference after treatment. Ultrasound-measured piriformis thickness results are shown in Table 3.

**Table 3:** Comparison of piriformis muscle thickness between the two groups before and after treatment.

Group	*n*	Pre-treatment thickness (mm)	Post-treatment thickness (mm)	Within-group *P*	Between-group *P* after treatment
Control group	30	17.01 ± 0.32	12.16 ± 0.25	<0.001	
Combined-treatment group	30	16.86 ± 0.27	9.04 ± 0.16	<0.001	<0.001

### 3.4 Clinical efficacy

After 2 weeks of treatment, the total effective rate was 46.7% (14/30) in the control group and 86.7% (26/30) in the combined-treatment group, indicating a higher overall clinical response in the combined-treatment group (Fisher exact *p* = 0.002). The cure rate was also higher in the combined-treatment group (10/30, 33.3%) than in the control group (6/30, 20.0%); however, the difference in cure proportion alone did not reach statistical significance when analyzed as cured versus non-cured (Fisher exact *p* = 0.382). The four-category clinical efficacy distribution differed significantly between groups (chi-square = 10.87, *p* = 0.012). Because total effective rate is a supportive categorical outcome and is less commonly used internationally than VAS or objective imaging outcomes, these findings were interpreted cautiously and in conjunction with the VAS and MSUS results. Clinical efficacy categories are summarized in Table 4.

**Table 4:** Comparison of clinical efficacy between the two groups.

Group	*n*	Cured	Markedly effective	Effective	Ineffective	Total effective rate	Fisher exact *p*-value for total effective rate
Control group	30	6	4	4	16	46.7%	
Combined-treatment group	30	10	8	8	4	86.7%	0.002

The combined-treatment group showed a significantly higher total effective rate and a more favorable four-category efficacy distribution than the control group. The cure rate was numerically higher in the combined-treatment group, but the cure-proportion comparison alone did not reach statistical significance. The total effective rate was analyzed as a supportive secondary outcome.

### 3.5 Safety analysis

No serious adverse events, including skin infection, subcutaneous hematoma, nerve injury, severe dizziness, nausea, or treatment discontinuation, occurred in either group. In the combined-treatment group, two patients experienced mild subcutaneous bruising and transient hip soreness after acupuncture; both events resolved spontaneously within 2 to 3 days without additional intervention. No adverse reactions were observed in the control group. The incidence of adverse events was low, and all events were mild and self-limiting.

## 4 Discussion

The present study found that both ESWT alone and Linggui Tanxue acupuncture combined with ESWT improved pain and reduced MSUS-measured piriformis muscle thickness after 2 weeks of treatment. Compared with ESWT alone, the combined-treatment regimen produced significantly greater reductions in VAS pain score and piriformis muscle thickness, indicating better short-term improvement in the main continuous outcomes. The combined-treatment group also showed a higher total effective rate and a more favorable four-category efficacy distribution than the control group. However, because the total effective rate is not widely used in international musculoskeletal research, the therapeutic advantage of the combined regimen should be interpreted primarily from the validated VAS pain outcome and the objective MSUS-measured piriformis thickness outcome, with categorical efficacy used only as supportive evidence.

The possible mechanisms of improvement may involve both functional and structural effects. PS is associated with piriformis muscle spasm, local inflammation, edema, fascial adhesion, and irritation or compression of the sciatic nerve (1, 2). ESWT may reduce pain through mechanical stimulation, modulation of peripheral nociceptive input, improvement of local microcirculation, and reduction of soft-tissue adhesion (3–5, 9). Acupuncture may further contribute to analgesia and muscle relaxation through local needling stimulation at tender or reactive points and by reducing local muscle tension (6, 7). The combination of acupuncture and ESWT may therefore produce complementary effects on pain reduction and local soft-tissue recovery, which is consistent with the greater reductions in VAS score and piriformis muscle thickness observed in the combined-treatment group.

MSUS was used in this study as an objective imaging tool to quantify piriformis muscle thickness. This is clinically relevant because pain intensity is subjective and may be influenced by expectation, treatment experience, and evaluator bias. Previous studies have suggested that ultrasound can be used to assess piriformis morphology and muscle thickness in patients with PS (11–14). To reduce measurement bias, pain scores were collected by blinded evaluators, and ultrasound measurements were performed by a senior ultrasonographer blinded to group allocation using the same anatomical scanning plane and measurement protocol. Nevertheless, a formal intra-observer or inter-observer reliability study was not conducted. Therefore, the ultrasound results should be interpreted as standardized single-assessor measurements rather than as independently validated reproducibility data.

The clinical efficacy analysis further supported the advantage of the combined-treatment regimen. Compared with ESWT alone, Linggui Tanxue acupuncture combined with ESWT produced a higher total effective rate and a more favorable four-level clinical efficacy distribution. The cure rate was also numerically higher in the combined-treatment group; however, the cure-rate comparison alone did not reach statistical significance, which may be related to the limited sample size. These findings suggest that the categorical efficacy outcomes are directionally consistent with the continuous outcomes, including VAS pain score and MSUS-measured piriformis thickness. However, the total effective rate should not be overemphasized as a definitive endpoint because it is less directly comparable across international musculoskeletal pain studies than validated pain scales and quantitative imaging measures.

This study has several limitations. First, it was conducted at a single center with a relatively small sample size, which may limit the generalizability of the findings. Second, no formal sample size calculation or statistical power analysis was performed before patient enrollment; therefore, the study may be underpowered for some secondary outcomes, and the precision of effect estimates is limited. Third, the study was not prospectively registered, and a predefined SAP was not established before study initiation. This reduces methodological transparency and may introduce risk of selective analysis or reporting bias, although the main outcomes and limitations are reported transparently. Fourth, the follow-up period was only 2 weeks. Therefore, no conclusions can be drawn regarding durability of treatment benefits, recurrence, or long-term functional outcomes. This is particularly important because PS may be persistent or recurrent. Fifth, because there is no universally accepted diagnostic gold standard for PS, some diagnostic uncertainty remains despite the use of clinical criteria and exclusion of alternative causes. Sixth, although pain assessment and ultrasound evaluation were performed by blinded assessors, participant blinding was not feasible because of the nature of the interventions. Seventh, formal ultrasound reliability testing was not performed. Finally, additional baseline variables such as body mass index, occupational exposure, affected side distribution, sitting duration, and physical activity level were not systematically collected and therefore could not be analyzed as potential confounders.

Despite these limitations, the study has strengths, including prospective enrollment, use of a control group, standardized treatment protocols, blinded assessment of pain and ultrasound outcomes, and the combination of subjective and objective measures. The findings are encouraging and suggest that Linggui Tanxue acupuncture combined with ESWT may offer additional short-term benefit compared with ESWT alone. However, larger multicenter randomized studies with prospective registration, predefined SAPs, formal sample size calculations, standardized diagnostic algorithms, ultrasound reliability assessment, and longer follow-up are required before definitive clinical recommendations can be made.

## 5 Conclusion

Linggui Tanxue acupuncture combined with ESWT may provide greater short-term pain relief and greater reduction in MSUS-measured piriformis muscle thickness than ESWT alone in patients with PS. The categorical total effective rate was higher in the combined-treatment group, but this supportive outcome should be interpreted cautiously. The combined regimen was generally well tolerated, with no serious adverse events observed. Because of the exploratory design, lack of prospective registration and predefined SAP, absence of formal sample size calculation, and short 2-week follow-up period, these findings require confirmation in larger prospectively registered multicenter trials with longer follow-up (1, 3–5, 11).

## Data Availability

The raw data supporting the conclusions of this article will be made available by the authors, without undue reservation.
