# Correction: Clinical efficacy of Linggui Tanxue acupuncture combined with extracorporeal shock wave therapy for piriformis syndrome: a prospective comparative clinical study

**DOI:** 10.3389/fmed.2026.1960827

**Published:** 2026-08-21

**Authors:** Chao Xue, Jie Ma, Longlong Wei, Songgang Liu

**Affiliations:** Qingdao Huangdao District Central Hospital, Qingdao, Shandong, China

**Keywords:** acupuncture, extracorporeal shock wave therapy, musculoskeletal ultrasound, pain, piriformis syndrome

Authors Chao Xue, Jie Ma, Longlong Wei and Songgang Liu were erroneously omitted as equal contributing authors.

The following Equal contribution statement was added to these authors: “These authors have contributed equally to this work and share first authorship.”

The original version of this article has been updated.

